# Applying Dimensionality Reduction Techniques in Source-Space Electroencephalography *via* Template and Magnetic Resonance Imaging-Derived Head Models to Continuously Decode Hand Trajectories

**DOI:** 10.3389/fnhum.2022.830221

**Published:** 2022-03-24

**Authors:** Nitikorn Srisrisawang, Gernot R. Müller-Putz

**Affiliations:** ^1^Institute of Neural Engineering, Graz University of Technology, Graz, Austria; ^2^BioTechMed Graz, Graz, Austria

**Keywords:** electroencephalography (EEG), magnetic resonance imaging (MRI), source localization, partial least squares regression, unscented Kalman filter, frontoparietal network, movement decoding

## Abstract

Several studies showed evidence supporting the possibility of hand trajectory decoding from low-frequency electroencephalography (EEG). However, the decoding in the source space *via* source localization is scarcely investigated. In this study, we tried to tackle the problem of collinearity due to the higher number of signals in the source space by two folds: first, we selected signals in predefined regions of interest (ROIs); second, we applied dimensionality reduction techniques to each ROI. The dimensionality reduction techniques were computing the mean (Mean), principal component analysis (PCA), and locality preserving projections (LPP). We also investigated the effect of decoding between utilizing a template head model and a subject-specific head model during the source localization. The results indicated that applying source-space decoding with PCA yielded slightly higher correlations and signal-to-noise (SNR) ratios than the sensor-space approach. We also observed slightly higher correlations and SNRs when applying the subject-specific head model than the template head model. However, the statistical tests revealed no significant differences between the source-space and sensor-space approaches and no significant differences between subject-specific and template head models. The decoder with Mean and PCA utilizes information mainly from precuneus and cuneus to decode the velocity kinematics similarly in the subject-specific and template head models.

## Introduction

The relation between the arm/hand movement and the brain has been one of the prominent fields of research in neuroscience because this knowledge can be extended to a brain-computer interface (BCI) (Wolpaw et al., 2002). The main goal of BCI is to improve the quality of life of people with limited body function after disease or injury. One of the goals is to restore motor function *via* direct control from the brain, e.g., the arm/hand function for people with paralyzed upper extremities. On the neuroscience side, the arm movement direction in two dimensions of non-human primates was studied and related to the neural activity in the motor cortex and parietal lobes by analyzing single neuronal activity (Georgopoulos et al., 1982; Kalaska et al., 1983). It was later extended to the 3D arm movement (Georgopoulos et al., 1988; Kettner et al., 1988; Schwartz et al., 1988; Caminiti et al., 1990). These studies paved the path towards decoding of the hand/arm movement kinematics in non-human primates (Wessberg et al., 2000; Carmena et al., 2003; Musallam, 2004; Lebedev, 2005; Li et al., 2009). Thereafter, invasive decoding of movement was also proven to be possible in humans *via* intracortical measurement (Hochberg et al., 2012, 2006; Collinger et al., 2013; Wodlinger et al., 2015; Bouton et al., 2016; Ajiboye et al., 2017; Willett et al., 2017) and electrocorticography (ECoG) (Schalk et al., 2007; Pistohl et al., 2008; Nakanishi et al., 2013; Wang et al., 2013; Hammer et al., 2016). Non-invasively, arm movement trajectories were decoded *via* magnetoencephalography (MEG) (Georgopoulos et al., 2005; Jerbi et al., 2007; Waldert et al., 2008; Yeom et al., 2013; Kobler et al., 2019a) and electroencephalography (EEG) (Bradberry et al., 2010; Lv et al., 2010; Ofner and Muller-Putz, 2012; Antelis et al., 2013; Úbeda et al., 2017; Korik, 2018; Kobler et al., 2018, 2020c; Martínez-Cagigal et al., 2020; Mondini et al., 2020). Despite the low signal-to-noise ratio in EEG, Bradberry et al. (2010) showed that the hand movement kinematics could be predicted from the low-frequency EEG. The possibility of hand movement kinematics decoding from EEG has been confirmed by several studies in both the executed (Lv et al., 2010; Ofner and Muller-Putz, 2012; Antelis et al., 2013; Úbeda et al., 2017; Kobler et al., 2020a,c; Martínez-Cagigal et al., 2020; Mondini et al., 2020), observing (Kobler et al., 2018, 2020c), and also imagery of the hand movement (Korik, 2018). These studies in humans suggested an involvement of the brain regions in the sensorimotor cortex and parietal lobes, which corresponded to the frontoparietal network (Culham and Valyear, 2006; Marek and Dosenbach, 2018).

On the other hand, non-invasive research in the BCI context has been commonly done in the sensor space, i.e., by directly using EEG signals. But another way emerged recently when the advancement of source localization techniques allowed us to infer cortical sources from non-invasive brain signals in real-time more accurately. For an extensive review of different source localization techniques, we recommend the readers to Hallez et al. (2007), Grech et al. (2008), He et al. (2018). In fact, some studies have already investigated the possibility of decoding brain activity in the source space *via* the source localization of the EEG (Qin et al., 2004; Shenoy Handiru et al., 2017; Xygonakis et al., 2018; Edelman et al., 2019; Li et al., 2021; Sosnik and Zheng, 2021; Srisrisawang and Müller-Putz, 2021). The source-space decoding has been primarily explored in the domain of classification: motor imagery classification (Qin et al., 2004; Xygonakis et al., 2018; Edelman et al., 2019), movement-related cortical potential (MRCP) classification (Li et al., 2021), arm directions classification (Shenoy Handiru et al., 2017). Most of the studies in the source-space classification reported an improved performance in comparison to the sensor space. On the other hand, in the domain of regression, a recent study (Sosnik and Zheng, 2021) on source-space decoding shows that the joint trajectories could be decoded. However, they reported a slightly lower correlation from the source-space decoding than the sensor-space decoding.

In this study, we would like to extend the investigation from our previous study (Srisrisawang and Müller-Putz, 2021) on the source-space decoding by improving the definition of the regions of interest (ROIs), as well as investigating the effect of incorporating the subject-specific information from magnetic resonance imaging (MRI). We hypothesized that the performance of source-space decoding could be improved when including the subject-specific information.

## Materials and Methods

### Participants

The dataset used in this analysis consisted of 15 participants collected from 14 non-disabled participants from 2 similar studies published before by our group. In the first study (Mondini et al., 2020), there are 10 participants (five male, five female, mean age: 27 ± 3.71 years old, at the time of measurement), and in the second study (Martínez-Cagigal et al., 2020), there are 5 participants (two male, three females, mean age: 28.2 ± 2.4 years old, at the time of the measurement). The experimental procedure was conformed to the declaration of Helsinki and was approved by the local ethics committee. Written informed consent was obtained from participants prior to the experiment. One participant was excluded due to signal quality problems, and another one participated in both studies. The data of this participant from two studies were treated as two separate participants.

### Experimental Paradigm and Dataset Description

**FIGURE 1:**
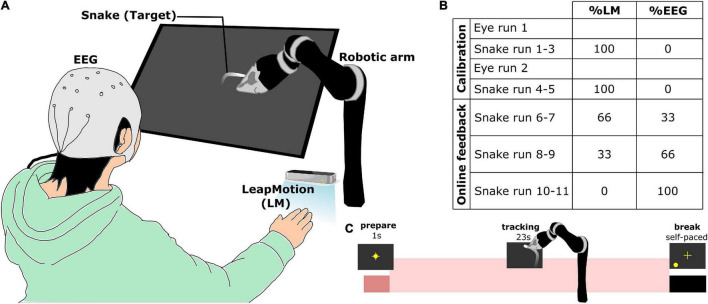
Experimental information (adapted from Mondini et al., 2020). **(A)** Overall experimental setup. Participants sat in front of a tilted screen while placing his/her right hand on the table under a LeapMotion (LM) system. **(B)** Mixing percentages of the control signals for the robotic arm across the whole experiment. **(C)** Temporal structure of a single trial during snake run. A trial began with the participant fixating his/her eyes on the cross for 1 s. The participant had to control the robotic arm to follow the snake target for 23 s.

The participants were sitting in a comfortable chair in front of a tilted monitor screen with a robotic arm (JACO, Kinova Robotics Inc., Canada) and a LeapMotion (LM) system (LeapMotion Inc., United States) that measures the kinematics of a hand (Figure 1A). The participants placed their right hand below the LM controller. There was a subtle difference in how the robotic arm was positioned between the two studies, but the effect can be neglectable. In the first experiment, the end effector of the robotic arm pointed directly to the screen. However, in the second experiment, the robotic arm held a small stick that pointed above the screen, which was less obstructive.

The experiment was divided into calibration and online experiment with feedback (Figure 1B, see also Martínez-Cagigal et al. (2020), Mondini et al. (2020) for more details). There were seven measurement runs from the calibration part: two eye artifact runs for recording EOG/EEG with specific eye movement to train an eye-artifact removal model. Thereafter, there are five snake runs, in which the participant was instructed to track the random snake trajectory. The data from EEG and LM from these five snake runs were used to train the decoding models, which provided the visual feedback later in another six snake runs in the online experiment with feedback. At the beginning of each trial, participants were asked to fixate their eyes on the yellow cross of the screen until the cross disappeared and they had to follow the snake target with their hand (Figure 1C). The control signals of the robotic arm were produced by mixing the movement kinematics from the LM system and the predicted movement kinematics from EEG at different ratios (33, 66, and 100% EEG control for snake run 6–7, 8–9, 10–11, respectively).

**FIGURE 2:**
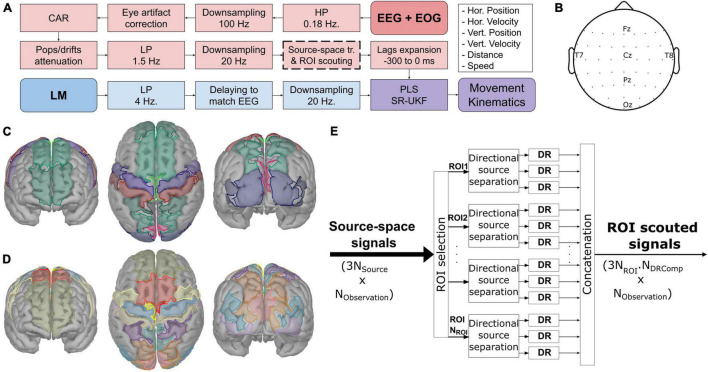
The general information of the experiment. **(A)** The common processing pipeline based on Martínez-Cagigal et al. (2020). Thick dashed lines indicate the additional part (source-space transformation and regions-of-interest scouting). **(B)** The common electrode layout from both studies. **(C,D)** The region-of-interest used with the ICBM152 template head model and the participant-specific head model from MRI, respectively, projected to the 15,000 voxels head model for visualization purposes. The ROIs were derived from Srisrisawang and Müller-Putz (2021) by further dividing each of the following regions into two subregions: superior frontal gyri, precentral gyri, postcentral gyri, superior parietal lobes, and occipital gyri. **(E)** The overview processes of scouting the signals in the region-of-interest and applying the dimensionality reduction (DR) techniques to produce the region-of-interest scouted signals.

The EEG signals were measured with 64-channel active EEG electrodes (actiCAP, *Brain Products GmbH, Germany*) connected to the biosignal amplifiers (BrainAmp, *Brain Products GmbH, Germany*) at 500 Hz. There were slight differences in the layout of the electrodes. However, only the 53 common electrodes were considered in this analysis, as shown in Figure 2B.

### Common Processing Pipeline

The processing was implemented *via* custom scripts on MATLAB R2019b (MathWorks Inc., MA, United States) based on the EEGLAB package (Delorme and Makeig, 2004). Prior to the calibration, the EEG was visually inspected to find any contaminations. The contaminated channels were removed and interpolated. The common processing pipeline is summarized in Figure 2A. The processes were divided into two branches: EEG + EOG, and LM branches, before being combined to produce the movement kinematics. The EEG signals were high-pass filtered with a cutoff frequency at 0.18 Hz, downsampled from 500 to 100 Hz. Then, they were corrected for any eye artifact contaminations using the sparse generalized eye artifact subspace subtraction algorithm (SGEYESUB) (Kobler et al., 2020b), re-referenced to the common average reference (CAR), corrected again for the pop and drifts artifact *via* the high-variance electrode artifact removal (HEAR) algorithm (Kobler et al., 2019b), low-pass filtered with a cutoff frequency of 1.5 Hz. At this stage, the contaminated trials were rejected according to their signal amplitude, joint probability, variance, and kurtosis before the remaining signals were downsampled to 20 Hz. After that, the signals were transformed into the source-space *via* a source localization technique (see below) and reduced according to the predefined ROIs. Thereafter, they got extended with 7 of their lags from −300 to 0 ms. The kinematic signals from LM were low-pass filtered with a cutoff frequency of 4 Hz, delayed, and downsampled to 20 Hz to match the EEG signals. Afterward, a partial least squares (PLS) regression model (Wold et al., 2001) and a square-root unscented Kalman filter (SR-UKF) (Van der Merwe and Wan, 2001) were trained with the processed signals from both branches. The combination of PLS and SR-UKF models produced the movement kinematics, directional and non-directional kinematics, namely, horizontal position *p*_*hor*_, horizontal velocity *v*_*hor*_, vertical position *p*_*ver*_, vertical velocity *v*_*ver*_, distance *d*, and speed *s*. All filters were second order infinite impulse response (IIR) Butterworth filters.

### Magnetic Resonance Imaging Acquisition and Processing

The participant-specific anatomical MRI images were acquired *via* a 3T MRI machine (Siemens Magnetom Vida, Erlangen, Germany) with a 20-channel head coil. A T1-weighted magnetization prepared rapid acquisition gradient-echo (MPRAGE) sequence was applied during the acquisition. The MRI images were acquired from six participants, making the total number of participants with MRI images of seven participants (because there was a participant that was in both studies). The cortical surface was extracted from the MRI images *via* a graphical user interface of the BrainSuite 19b (Shattuck et al., 2001; Shattuck and Leahy, 2002). The cortical surface was automatically labeled according to the USCBrain atlas (Joshi et al., 2020).

### Source-Space Transformation

In order to transform the sensor-space signals into the source-space signals, a source localization was performed using the Brainstorm package (Tadel et al., 2011) and OpenMEEG (Gramfort et al., 2010).

The EEG generation can be modeled as


(1) X=G⁢J+n

where *X* is an *N*_*Channel*_×*N*_*Observation*_ matrix containing the sensor-space EEG signals, *G* is an *N*_*Channel*_×*N*_*Source*_ gain matrix, *J* is an *N*_*Source*_×*N*_*Observation*_ matrix with signals from the true cortical sources, and *n* is an *N*_*Channel*_×*N*_*Observation*_ matrix of additive noise.

The forward problem was modeled using a boundary element method (BEM). The BEM modeled the brain with three compartments with different electrical conductivity properties: brain, skull, and scalp. The conductivity of each compartment was assumed to be homogeneous across the compartment. The conductivity was set to (0.41, 0.02, and 0.47) for scalp, skull, brain, respectively, according to the meta-analysis results (McCann et al., 2019). The number of source components was set to 5,000 cortical sources. The orientation of each source was not fixed, resulting in 3 directional source components per cortical source. The inverse problem was solved *via* the standardized low-resolution brain electromagnetic (sLORETA) method (Pascual-Marqui, 2002). The noise covariance was computed from the artifact corrected signals from the eye runs.

At the beginning of the experiment, the electrode positions were acquired (ELPOS, Zebris Medical Gmbh, Germany). This information was used to co-register the electrodes onto the cortical surface. In the template head model, the cortical surface came from the ICBM152 template (Collins et al., 1999; Fonov et al., 2011, 2009), while for the participant-specific head model, the cortical surface extracted from the MRI images was used.

### Regions of Interest Scouting and Dimensionality Reduction

The idea of ROI scouting is to reduce the number of signals from the source-space transformation because the number of signals increases by 300 times from about 50 signals to 15,000 signals (we have 5,000 cortical sources with 3 directional source components per cortical source). However, even considering only signals in the defined ROIs would not help much as the number of signals would still be in the range of thousands of signals. Hence, the dimensionality reduction (DR) techniques were implemented to further reduce the number of signals.

The ROIs were defined based on the Mindboggle atlas (Klein et al., 2017) for the template head model (see Figure 2C), and for the subject-specific head model, the ROIs were based on the USCBrain atlas (see Figure 2D; Joshi et al., 2020). The selected ROIs were chosen according to the frontoparietal network (Culham and Valyear, 2006; Marek and Dosenbach, 2018) and the source localization results from the previous EEG hand trajectory decoding studies (Kobler et al., 2019a; Martínez-Cagigal et al., 2020; Mondini et al., 2020). The selected ROIs were cuneus (CU), paracentral (PCL), postcentral (PoCG), precentral (PreCG), precuneus (PCU), superior frontal (SFG), occipital, and superior parietal (SPL) regions of the brain, according to the previous study (Srisrisawang and Müller-Putz, 2021). However, we further divided SFG into anterior/posterior (aSFG, pSFG); PreCG into medial/lateral (mPreCG, lPreCG); PoCG into medial/lateral (mPoCG, lPoCG); SPL into anterior/posterior (aSPL, pSPL); occipital gyrus into superior/inferior (SOG, IOG) and in the case of subject-specific MRI, middle occipital gyrus (MOG) were also included to cover the same area as in the template head model (see Figures 2C,D). Afterward, several DR techniques were applied after ROI scouting. Three techniques were chosen: computing the mean (Mean), principal component analysis (PCA), and locality preserving projections (LPP) (He and Niyogi, 2004).

The process of reducing the signals is visualized in Figure 2E. After projecting the sensor-space EEG signals onto the source space, the signals were scouted according to the ROIs and in each ROI, the directional source components were separated. The DR technique was applied for each directional source component of each ROI separately, resulting in *N*_*feat*_ = 3*N*_*ROI*_⋅*N*_*DRComp*_ features, where *N*_*DRComp*_ indicates the number of components retained with DR.

#### Computing the Mean

This process was done by computing the mean value across all sources within each ROI for each time step. This produced only a representative signal per directional source component in each ROI, *N*_*DRComp*_ = 1.

#### Principal Component Analysis

The idea of PCA is to project observations into a lower dimension using linear combinations that maximally explains the variance of observations. The number of retained components was varied, where *N*_*DRComp*_ ∈ [1,2,4,8,16].

#### Locality Preserving Projections

Similarly, LPP (He and Niyogi, 2004) linearly maps observations into a lower dimension. However, instead of finding the subspace that maximally explains the variance as in PCA, LPP tries to find the subspace in the lower dimension that retains the structural information across observations. The first step of LPP is to form an adjacency matrix representing the structural information across observations. After that, the generalized eigenvector problem was solved based on this adjacency matrix. The number of retained components was also varied, where *N*_*DRComp*_ ∈ [1,2,4,8,16]. The adjacency matrix was formed *via* the *k*-nearest neighbor algorithm with *k=5*, and the weight between each pair of observations was computed by the heat kernel with *t* = 5. These *k* and *t* are different quantities than defined elsewhere in this study.

The processes of source-space transformation and the ROI scouting can be written in an equation as


(2) $$y_{t}=U\,K\,x_{t}$$

where *x_t_* is a column vector containing the sensor-space EEG signal at time *t* from *X*, *K* is an *N*_*Source*_×*N*_*Channel*_ kernel matrix resulting from the source localization, *U* is an *N*_*feat*_×*N*_*Source*_ scouting matrix representing the processing of ROI scouting and DR, *y_t_* is an *N*_*feat*_×1 matrix containing the reduced source-space signals at time*t*.

### Decoding Model

The directional movement kinematics at time *t*, *z_t_*, were extracted from the LM signals


(3) $$z_{t}=\left[p_{h\,o\,r,t};v_{h\,o\,r,t};p_{v\,e\,r,t};v_{v\,e\,r,t}\right]$$

and then extended with the non-directional movement kinematics as


(4) $${\tilde{z}}_{t}=e\,x\,t\,\left(z_{t}\right)=\left[p_{h\,o\,r,t};v_{h\,o\,r,t};p_{v\,e\,r,t};v_{v\,e\,r,t};d_{t};s_{t}\right]$$

where dt=ph⁢o⁢r,t2+pv⁢e⁢r,t2 and st=vh⁢o⁢r,t2+vv⁢e⁢r,t2.

The reduced source-space signals were extended with multi lags ranging from 0 to −300 ms (equivalently, 0th lag to −6th lag at 20 Hz). Resulting in a column vector of the extended source-space signals, ${\tilde{y}}_{t}$, with a size of 6*N*_*feat*_×1 as


(5) $${\tilde{y}}_{t}=\left[y_{t};y_{t-1};\ldots ;y_{t-6}\right]$$

The *z*-score of *z_t_* and ${\tilde{y}}_{t}$ was computed. The PLS regression was trained *via* SIMPLS algorithm (de Jong, 1993). The SIMPLS algorithm finds the latent space that explains the most variance of the cross-covariance matrix between the ground truth movement kinematics, *z*, and the extended reduced source-space signals, $\tilde{y}$. The PLS regression weight matrix, *W*, projects the signals into a new latent space which can be written as


(6) $${\tilde{e}}_{t}=W\,{\tilde{y}}_{t}$$

where *W* is *N*_*PLSComp*_×6⋅*N*_*feat*_ matrix that projects the extended reduced source-space signals into the latent space. *N*_*PLSComp*_ was chosen separately for each participant to retain 95% of the data covariance. After applying PLS to the extended source-space signals, a UKF (Wan and Van Der Merwe, 2000) model was applied to predict the movement kinematics.

The UKF model can be written as follows


(7) $$z_{t+1}=F\,z_{t}+q_{t}$$

(8) $${\tilde{e}}_{t+1}=h\,\left(z_{t+1}\right)+r_{t}$$

(9) $$h\,\left(z_{t+1}\right)=H\,e\,x\,t\,\left(z_{t+1}\right)$$

where *F* is a state transition matrix that relates the state (or in this case the movement kinematics) between observations at time step *t* and t+1, *q_t_* is a state transition error, *h*(.) is a nonlinear mapping function, *r_t_* is an observation error, and *H* is an observation matrix. Equations 7, 8 are called a process equation and a measurement equation. The nonlinear mapping was included only in the measurement equation but not in the process equation.

The UKF model was then initialized as follows (Martínez-Cagigal et al., 2020)


(10) $$F=\Sigma_{z_{t},z_{t-1}}\,\Sigma_{z_{t},z_{t-1}}^{-1}$$

(11) $$Q=\Sigma_{\epsilon_{q},\epsilon_{q-1}},\epsilon_{q}=F\,z_{t-1}-z_{t}$$

(12) $$H=\Sigma_{{\tilde{e}}_{t},z_{t-1}}\,\Sigma_{z_{t-1},z_{t-1}}^{-1}$$

(13) $$R=\Sigma_{\epsilon_{r},\epsilon_{r}},\epsilon_{r}=h\,\left(z_{t}\right)-{\tilde{e}}_{t},$$

where Σ_*a*,*b*_ indicates the covariance matrix between *a* and *b*, *A*^−1^ indicates an inverse of a matrix *A*, ϵ_*q*_ and ϵ_*r*_ indicate the state transition error and the observation error, respectively. In the general form of the UKF (Wan and Van Der Merwe, 2000), the algorithm has to compute a square root of a matrix frequently, which is computationally intensive. The SR-UKF extends from the UKF by avoiding direct computation of this matrix square root *via* the Cholesky factor updating of the square root (Van der Merwe and Wan, 2001).

In the case of sensor-space decoding, the processes of source-space transformation and the ROI scouting were skipped. After the same processing pipeline and the lag expansion, the sensor-space signals were fed directly into the PLS regression and the SR-UKF models to predict movement kinematics.

### Performance Evaluation

The decoding models trained with the data from the 0% EEG measurement block (equivalent to 100% LM) were then applied to the data from 33, 66, and 100% EEG. However, in the 0% EEG, we used the leave-one-trial-out training scheme, keeping one trial as the test data and then using the rest as the training data. It was repeated as many times as the number of trials in the 0% EEG blocks.

The following metrics were used to compare the performance between the source-space and the sensor-space approaches, namely, Pearson’s correlation, signal-to-noise ratio (SNR), and decoded-signal-to-signal ratio (DSSR). An SNR can be expressed as


(14) $$S\,N\,R\,\left(z,\hat{z}\right)=10\,l\,o\,g_{10}\,\left(\frac{v\,a\,r\,\left(z\right)}{M\,S\,E\,\left(z,\hat{z}\right)}\right)$$

which is the ratio of the variance of the ground truth kinematics over the mean squared error between the predicted and the ground truth kinematics. A DSSR can be expressed as


(15) $$D\,S\,S\,R\,\left(z,\hat{z}\right)=10\,l\,o\,g_{10}\,\left(\frac{v\,a\,r\,\left(\hat{z}\right)}{v\,a\,r\,\left(z\right)}\right)$$

which is the ratio between the predicted kinematics variance and the ground truth kinematics variance. The DSSR quantifies the amplitude mismatch between the predicted and the ground truth kinematics. A negative value of the DSSR indicates that the amplitude of the predicted kinematics is smaller than the ground truth kinematics and vice versa. The best case is when DSSR is at 0 dB, indicating that the amplitude of the predicted and the ground truth kinematics match (Kobler et al., 2020c). We applied multiple comparisons with Bonferroni method to compare between approaches.

First, the number of DR components (*N*_*DRComp*_) was varied for PCA, LPP from 1, 2, 4, 8, and 16 components. The correlations were compared to find the optimum *N*_*DRComp*_ for both the approach with ICBM152 template head model (*PCA* and *LPP*) and the one with MRI (*PCA* + *MRI* and *LPP* + *MRI*). The group-level correlations were first grouped across the 0–100% EEG measurement runs. Then they were grouped into position (horizontal and vertical position), velocity (horizontal and vertical velocity), and magnitude (distance and speed). In the case of MRI, 7 participants with available MRI were considered, and in the case of ICBM152, 14 participants were considered.

Second, the optimum *N*_*DRComp*_ was used to compare across different approaches for sensor-space (*Sensor*) and source-space approaches. There are six variations of the source-space approaches depending on the DR function and the head model, namely: *Mean*, *PCA*, *LPP*, *Mean* + *MRI*, *PCA* + *MRI*, *LPP* + *MRI*. However, only the same seven participants with available MRI were considered for all approaches in this comparison.

### Source-Space Decoding Patterns

In order to visualize the decoding pattern, the subject-specific decoding pattern was obtained as follows


(16) $$A=\frac{1}{g}\,\Sigma_{\tilde{y}}\,W\,\Sigma_{\tilde{z}}^{-\frac{1}{2}}$$

The PLS regression weight matrix *W* was transformed into the interpretable decoding pattern by multiplying it with the covariance matrix of the extended source-space signals $\Sigma_{\tilde{y}}$ and the inverse of the covariance matrix of the extended movement kinematics $\Sigma_{\tilde{z}}$ according to Haufe et al. (2014). The decoding pattern was then rescaled by a matrix square root of $\Sigma_{\tilde{z}}$, in order to interpret the decoding pattern in voltages (Ofner et al., 2017; Mondini et al., 2020). The decoding pattern was rescaled by the reciprocal of the subject-specific global field power *g* (GFP) to reduce the different scaling across participants (Kobler et al., 2018). The subject-specific GFP was computed by randomly selecting a time point, averaging over trials, and computing the standard deviation across ROIs. This process was repeated for 10,000 permutations. The median of the standard deviation over permutations was selected for the subject-specific GFP (Kobler et al., 2020c). Since the decoding was done directly in the source space, there was no need to project the weight matrix onto the source space. We separated the absolute value of the weights of the decoding pattern for each lag of each ROI and then assigned them to the source accordingly (i.e., the sources in the same ROI have the same weight). We assigned value 0 to the sources outside ROI, resulting in these regions visualized in gray. For the ICBM152 variations, the group level decoding patterns were computed across participants and then projected onto the 15,000 voxels head model. For the MRI variations, the subject-specific decoding patterns from the subject-specific head model were first projected onto the ICBM152 head model with 15,000 voxels. Then the group level decoding patterns were computed. Only the first component was considered for visualization in the case of *PCA* and *LPP* variations.

## Results

### Choosing Optimal Component Number for Principal Component Analysis and Locality Preserving Projections

**FIGURE 3:**
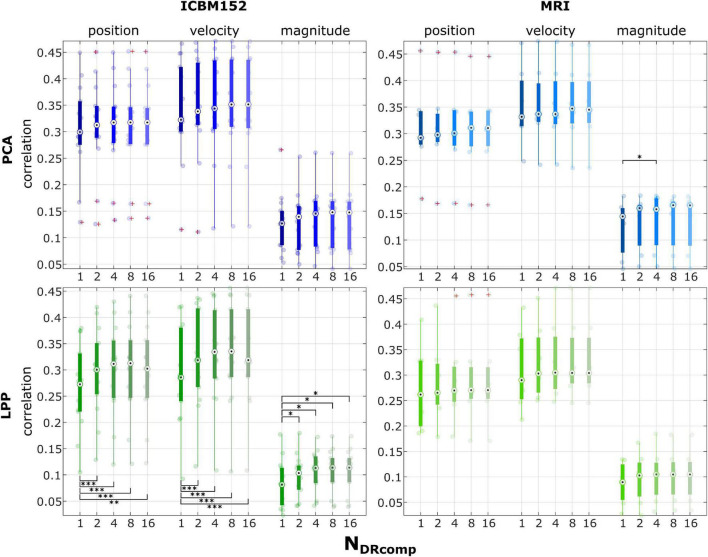
Group level correlations with the ICBM152 template head model or the subject-specific MRI images, with varying numbers of components from the PCA and LPP at 1, 2, 4, 8, 16 components. The correlations across different measurement runs (0–100% EEG) were grouped as well as combined into position (horizontal and vertical position), velocity (horizontal and vertical velocity), and magnitude (distance and speed). The red pluses indicated the outliers. The number of asterisk indicates the significant level *via* Bonferroni method for multiple comparisons, * *p* < 0.05, ^**^ *p* < 0.01, and ^***^ *p* < 0.001.

The results from varying the DR components (*N*_*DRComp*_) for *PCA*, *PCA* + *MRI*, *LPP*, and *LPP* + *MRI* are summarized in Figure 3. In all of the *PCA and LPP* variations, the correlation increased as the number of components increased and then reached a plateau or dropped a little (for example, in position and velocity in *LPP*). In the case of *PCA*, the highest correlations can be found with eight components for position (0.31 ± 0.09, mean ± SD), velocity (0.35 ± 0.09), and magnitude (0.13 ± 0.06). For *PCA* + *MRI*, the highest correlations can be found in 16 components for position (0.31 ± 0.08), velocity (0.35 ± 0.07) but found in four components for the magnitude (0.14 ± 0.05). For *LPP*, the highest correlations can be found with eight components for position (0.30 ± 0.09), velocity (0.33 ± 0.10), and magnitude (0.11 ± 0.04). For *LPP* + *MRI*, the highest correlations can be found with eight components for position (0.30 ± 0.09), velocity (0.33 ± 0.08), and magnitude (0.10 ± 0.05). The multiple comparisons with Bonferroni method indicated only statistically significant differences in the correlation of magnitude between 1 and 4 components for *PCA* + *MRI*. In *LPP*, the statistically significant differences could be seen between 1 component and the rest, in all kinematics. In most cases, the highest correlation could be achieved at eight components.

**FIGURE 4:**
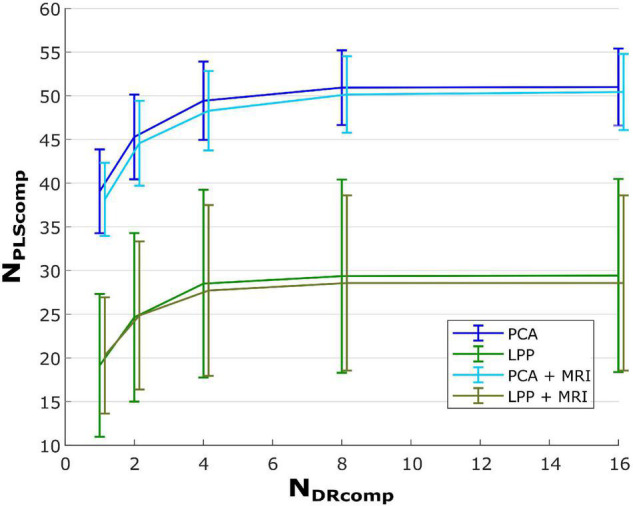
Plot of PLS latent components over the number of DR components that explain 95% of the variance. The bar indicates ±SD of the corresponding case.

Furthermore, Figure 4 shows line plots of the number of DR components (*N*_*DRComp*_) and the number of the latent components of the regression (PLS) explaining 95% (*N*_*PLSComp*_) of the variance for *PCA*, *PCA* + *MRI*, *LPP*, and *LPP* + *MRI*, respectively. The number of PLS latent components increased as the number of DR components increased but saturated after eight DR components, similar to the correlations observed in Figure 3. Two observations can be made here. First, the *PCA* variations showed a higher number of PLS latent components than *LPP* variations and, second, the MRI variation showed a slightly lower number of PLS latent components than the ICBM152 variation. At eight DR components, the averaged number of PLS components for *PCA*, *PCA* + *MRI*, *LPP*, and *LPP* + *MRI* were 50.93 ± 4.26 (mean ± SD), 50.14 ± 4.37, 29.36 ± 11.06, and 28.57 ± 10.03 components, respectively. When the DR components increased to 16, the average number of PLS components increased only slightly by 0.07, 0.07, and 0.29 for *PCA, LPP*, and *PCA* + *MRI*, respectively, but stayed the same for *LPP* + *MRI*. Due to the saturation of both the correlations and the number of PLS components, the optimal number of DR components for all four approaches was chosen to be eight DR components.

### Comparing Across All Approaches

**FIGURE 5:**
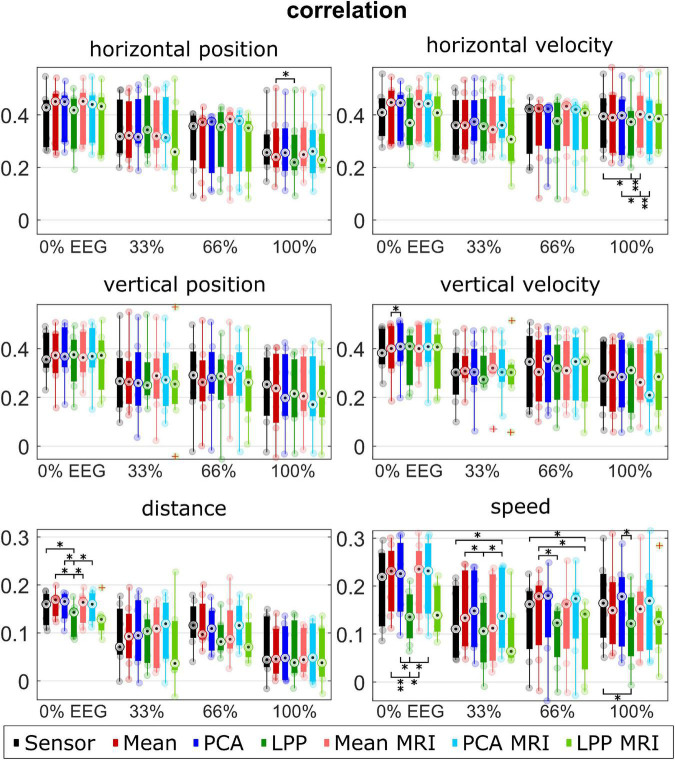
Group level correlations for each kinematic, measurement run, approach. Colors represent different approaches. Only seven participants with measured MRI were considered in the plot for the *Sensor, Mean, PCA*, and *LPP*. Each dot represents the mean correlation of each participant. The red pluses indicated the outliers. The number of asterisk indicates the significant level from Bonferroni method for multiple comparisons, *p < 0.05, ^**^p < 0.01, and ^***^p < 0.001.

**FIGURE 6:**
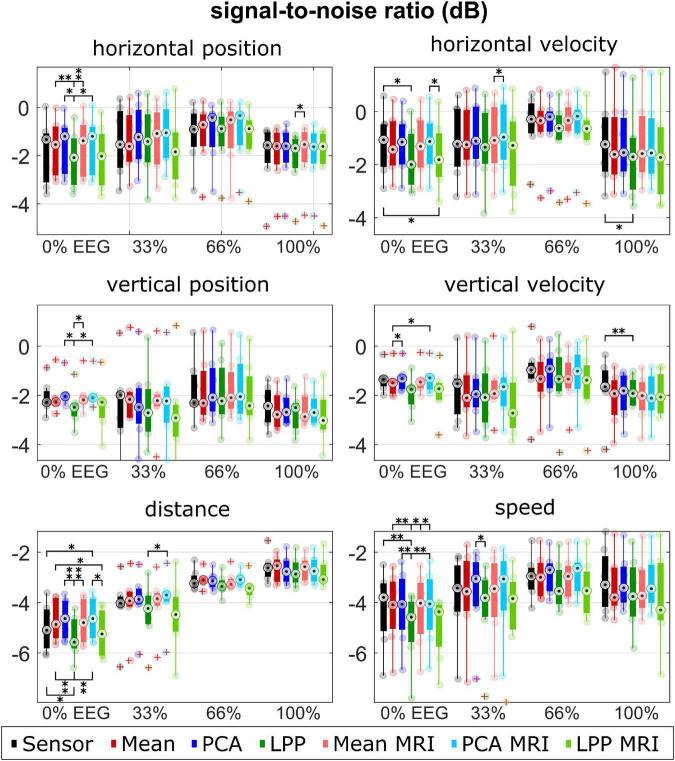
Group level SNRs for each kinematic, measurement run, approach. Colors represent different approaches. Only seven participants with measured MRI were considered in the plot for the *Sensor, Mean, PCA*, and *LPP*. Each dot represents the mean correlation of each participant. The red pluses indicated the outliers. The number of asterisk indicates the significant level from Bonferroni method for multiple comparisons, *p < 0.05, ^**^p < 0.01, and ^***^p < 0.001.

**FIGURE 7:**
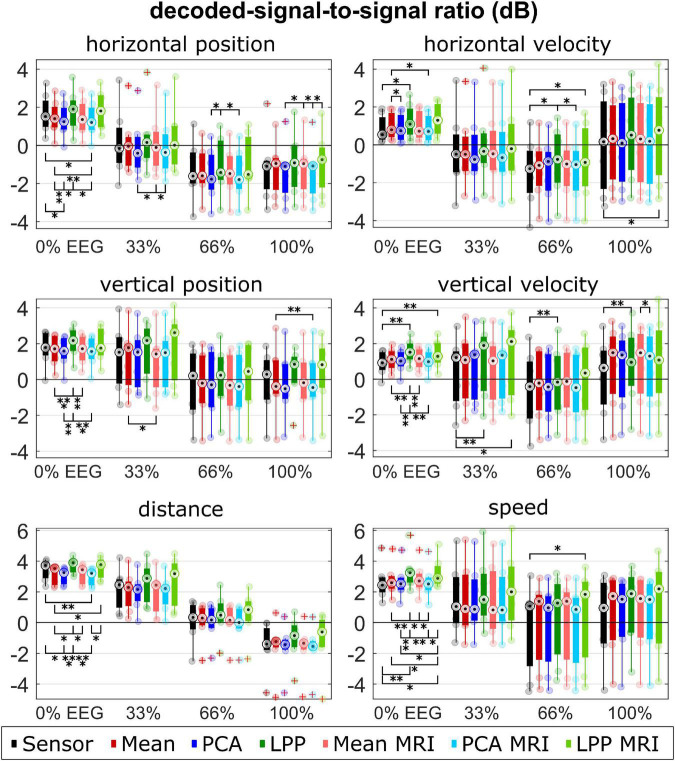
Group level DSSRs for each kinematic, measurement run, approach. Colors represent different approaches. Only seven participants with measured MRI were considered in the plot for the *Sensor, Mean, PCA*, and *LPP*. Each dot represents the mean correlation of each participant. The red pluses indicated the outliers. The number of asterisk indicates the significant level from Bonferroni method for multiple comparisons, *p < 0.05, ^**^p < 0.01, and ^***^p < 0.001.

The performance metrics are summarized in Figures 5–7 for each type of metric, kinematic, and measurement run (i.e., % EEG). Each dot represents the average metric of each participant. The boxplot and the dots were color-coded according to each approach: black for *Sensor*, red for *Mean*, blue for *PCA*, and green for *LPP*. In the case of the source-space approaches, the intensity of the color indicates whether the approach was with the ICBM152 head model (darker tone) or with the subject-specific head model from MRI (lighter tone).

Figure 5 visualizes the median correlations. Every approach indicated a similar range of the median correlations at around 0.2–0.4 for the directional kinematics and around 0.05–0.2 for the non-directional kinematics. We observed similar trends across different measurement runs and kinematics: *Mean, Mean* + *MRI* (red and light red), *PCA*, and *PCA* + *MRI* (blue and light blue), generally indicating a slightly higher correlation than *Sensor*. While *LPP* and *LPP* + *MRI* (green and light green) performed inconsistently compared to *Sensor*, it sometimes showed on-par or higher correlation (e.g., horizontal position at 33% EEG) but more commonly showed lower correlations than *Sensor*. The lower correlations of the *LPP* variations compared to the rest could be observed clearly in the 0% EEG measurement runs with speed kinematics, where the mean correlation of *Sensor* was at 0.20 ± 0.08, but the mean correlation of *LPP* was at 0.14 ± 0.05. We also observed the declining trend among approaches in all kinematics over measurement runs. Similar correlations between the ICBM152 variations and MRI variations could be observed for the same DR technique. The multiple comparisons showed significant differences between *LPP* to *Sensor*, *Mean*, *Mean* + *MRI, PCA*, and *PCA* + *MRI* mostly in the non-directional kinematics (distance 0% EEG; speed 0, 33, 66, and 100% EEG).

Similarly, the median SNRs (Figure 6) were similar for all approaches (around −2 to −1 dB for the directional kinematics and −5 to −3 dB for the non-directional kinematics). There was an increasing trend for all approaches of the median SNR over measurement runs, which could be observed clearly in the non-directional kinematics. *PCA*, *PCA* + *MRI* showed slightly higher SNR than *Sensor* in most cases, while *Mean* and *Mean* + *MRI* showed slightly lower SNR than *Sensor* in some cases. *LPP* and *LPP* + *MRI*, on the other hand, showed almost always lower SNR than *Sensor* and *Mean* and *PCA* variations. The significantly lower SNR in *LPP* than the other approaches could be seen dominantly in horizontal position 0% EEG, vertical position 0% EEG, distance 0% EEG, and speed 0% EEG.

For DSSR, the median DSSRs (Figure 7) were also in the same range for all approaches across different measurement runs and kinematics (around −2 to 2 dB for the directional kinematics and −2 to 4 dB for the non-directional kinematics). There was a decreasing trend observed across different measurement runs. Both *LPP* and *LPP* + *MRI* indicated almost always higher DSSR than other approaches. *PCA* and *PCA* + *MRI* showed, however, lower DSSR in most cases compared to *Sensor* and the rest. The statistical tests indicated significant differences between *LPP* and the other approaches, mainly in the 0% EEG measurement runs for all kinematics.

**FIGURE 8:**
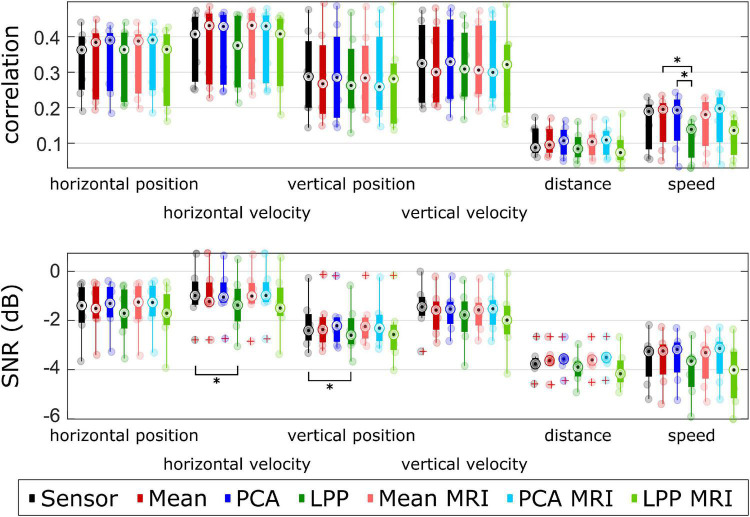
Group level metrics for each kinematics. Each dot represents an averaged metric across measurement runs for each participant. Different approaches were color-coded. The corresponding metrics for each participant were averaged across measurement runs. The red pluses indicated the outliers. The number of asterisk indicates the significant level from Bonferroni method for multiple comparisons, *p < 0.05, ^**^p < 0.01, and ^***^p < 0.001.

The results were further summarized in Figure 8 and Supplementary Table 1, where the performance metrics (excluding DSSR) were grouped by averaging the metrics of each participant across measurement runs. We noticed three general trends for every approach across kinematics in both the correlation and SNR. First, the decoding models could decode the directional kinematics better than the non-directional ones. Second, the decoding models could decode horizontal kinematics better than vertical ones. The last general trend was that the decoding model could decode the velocity kinematics slightly better than the position kinematics. We found statistically significant differences only between *LPP* to *Mean* and *LPP* to *PCA* in the case of speed correlation and between *Sensor* to *LPP* in SNR for the horizontal velocity and vertical position. We then compared an improvement in terms of correlations (see Supplementary Table 1) and found the highest improvement in comparison to *Sensor* mainly in *PCA* + *MRI*, which showed the highest improvement over *Sensor* in the horizontal position (improved by 0.028), distance (improved by 0.022), and speed (improved by 0.008). In terms of SNR, *PCA* + *MRI* showed the highest improvement in horizontal velocity (improved by 0.004 dB), distance (improved by 0.263 dB), and speed (improved by 0.125 dB). The average position correlations were 0.310 and 0.311 while the average velocity correlations were 0.349 and 0.353 and the average non-directional correlations were 0.132 and 0.136 for *Sensor* and *PCA* + *MRI*, respectively. Other observations were that in most cases within the same DR technique, the subject-specific head model showed higher correlations and SNRs in comparison to the template head model. However, we found no statistically significant differences. For *LPP* variations, the inclusion of the subject-specific head model leads to lower performance.

### Source-Space Decoding Patterns

**FIGURE 9:**
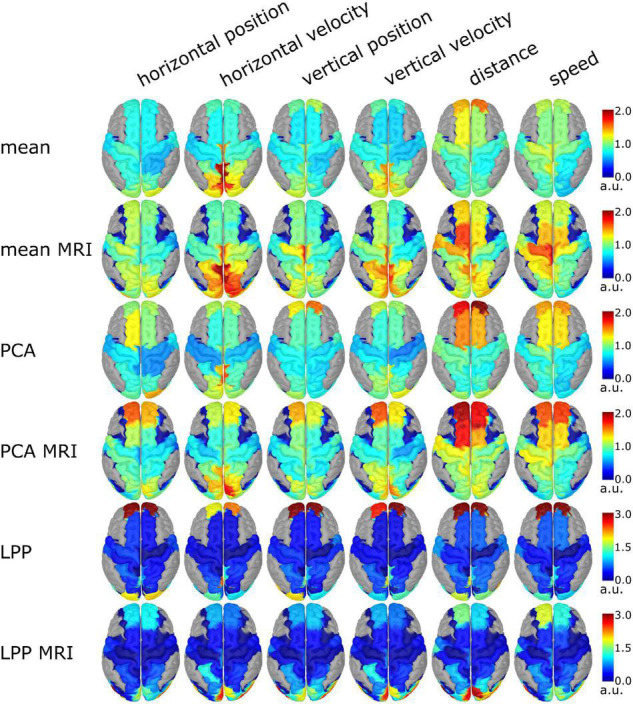
Group level source-space decoding pattern at lag 0 of every movement kinematic. The color represents the absolute intensity of the activity. The gray area indicated the brain region outside of the ROIs. In the case of *PCA* and *LPP variations*, only the decoding pattern of the first component was visualized.

The group level source-space decoding patterns of the 0th lag are summarized in Figure 9. Similar patterns could be observed between ICBM152 and MRI with the same DR technique. Both, *Mean* variations and *PCA* variations showed the absolute decoding patterns in the same range from 0 to 2, but *LPP* variations showed a much wider range from 0 to 8, but in Figure 9, we limited it to 0 to 3 to make the figure more interpretable. Among different approaches, the activity in PCU, cuneus (CU), and superior parietal lobes (SPL) could be seen dominantly in the velocity kinematics. In the case of position, less prominent activities were observed. For *PCA* and *Mean* variations, the dominant patterns are in the anterior part of superior frontal gyri (aSFG) and the weaker activity in the medial part of the pre- and postcentral gyri (mPreCG, mPoCG) for the non-directional kinematics, especially in the distance. In *LPP*, the strong activities from the anterior part of the superior frontal gyri (aSFG) dominated the contribution from other areas (such as PCU, and superior occipital gyri, SOG). Similar decoding patterns could be seen in *LPP* across movement kinematics. In *LPP* + *MRI*, the decoding patterns were similar to *Mean* and *PCA* variations but with dominating activities in CU and SOG.

## Discussion

We studied the hand trajectory decoding based on EEG but instead of utilizing the low-frequency EEG signals as in the previous studies (Kobler et al., 2018; Martínez-Cagigal et al., 2020; Mondini et al., 2020), we utilized the source-space signals obtained *via* the projection of the low-frequency EEG signals onto the source space. We proposed several source-space decoding approaches with three DR techniques (*Mean, PCA*, and *LPP*) and two head model variations (ICBM152, subject-specific MRI), resulting in six source-space approaches: *Mean, PCA, LPP*, and *Mean* + *MRI. PCA* + *MRI*, and *LPP* + *MRI*. These source-space approaches were compared to the sensor-space approach (*Sensor*).

First, the number of DR components (for *PCA, LPP*, and *PCA* + *MRI, LPP* + *MRI*) for each ROI was varied and compared. The number of DR components was chosen to eight components due to the saturation of both the correlations and the information (represented as the number of PLS components). As the number of DR components increased, the PLS model could utilize more information, leading to the increasing number of the PLS component. However, the saturation of the number of PLS components indicated that the additional information was not helpful in explaining the movement kinematics. We also observed that the number of PLS components of the *LPP* variations was always lower than the *PCA* variations, as well as that the number of the PLS components in the MRI head model was slightly lower than the ICBM152 template head model. For the first effect, we hypothesized that this is due to the different optimization criteria between *PCA* and *LPP*, where the former tries to maximize the explained variance of the data while the latter tries to retain the neighborhood information between observations. However, the neighborhood information contributed less useful information to the decoding model than the information retained with *PCA*. For the second effect, this could be due to the difference in the definition of the ROIs or due to the difference between the subject-specific head model and the ICBM152 template head model, though this effect resulted in only a slightly higher number of PLS components in the ICBM152 template head model than the subject-specific MRI.

Afterward, the *PCA* and *LPP* variations were compared to the other approaches by selecting eight DR components. We observed that the decoding correlations decreased over measurement runs in all approaches while the SNRs improved over the measurement runs. In the case of DSSR, the decoding model initially overestimated the amplitude of kinematics, but then the amplitude of the predicted kinematics became smaller over the measurement runs. The interpretation of the DSSR was difficult to comprehend because the quality of the DSSR depends on how close it is to 0 dB, in contrast to correlation and SNR, in which the higher it is, the better. According to the questionnaire analysis in Mondini et al. (2020), the participants perceived less control of the robotic arm over time, which also reflected the trends in the correlations and SNRs. The cause of these changes over the measurement runs could not be concluded whether this was because of the increased mental load due to the % EEG or the mental fatigue over time as the experiment progressed. Additional experiments might be needed in order to disentangle these factors.

Then, to ease the comparison between approaches, we averaged the correlations and SNRs for each participant across different % EEG. The DSSR was excluded because averaging the DSSR around 0 (see Figure 7) would result in numbers close to 0, which was not so useful for comparison. Across different approaches, we observed that the correlations of the velocity were higher than the position. A similar observation could also be seen in the non-directional kinematics, such that the speed showed slightly higher correlations than the distance. The same phenomena were already reported in Martínez-Cagigal et al. (2020), Mondini et al. (2020). This phenomenon, however, was not observable in some works that simultaneously decode the position and velocity kinematics. For example, in Hammer et al. (2016) and Kobler et al. (2020c), they reported correlations of the position and the velocity in a similar range and even contradicted the result in Ofner and Muller-Putz (2012) and Úbeda et al. (2017), where they reported higher position correlations than the velocity. We hypothesize that this discrepancy could be due to the differences in the experimental setup because of the lack of the robot arm in Kobler et al. (2020c), the lack of feedback in Ofner and Muller-Putz (2012), or the different tasks in Hammer et al. (2016) and Úbeda et al. (2017). Furthermore, the correlations of the horizontal components of the kinematics were generally higher than the vertical components. This phenomenon was also reported in Martínez-Cagigal et al. (2020), where they hypothesized that it was due to the tilted screen in the experiment that caused the mismatch between the actual vertical distance that the participant had to move and the vertical distance perceived. Similar observations could also be seen in the SNRs (Figure 8, lower part), where the velocities indicated higher SNRs than the positions, the speed indicated higher SNRs than the distance, and the horizontal components indicated higher SNRs than the vertical components. By comparing the metrics of each approach, we see that all of them performed in a similar range. *LPP* variations showed the lowest correlations and SNRs, while *Mean* and *PCA* variations showed higher correlations and SNRs than *Sensor* in most cases. It was reported in Li et al. (2021) that applying the *LPP* to the source-space EEG signals improved the true positive rate in the MRCP detection over the sensor-space EEG signals. While their processing pipeline was similar to our approach, our results suggested lower decoding performance in *LPP* than the sensor-space approach and even to the other source-space approaches. These lower decoding performances, in some cases, were significantly lower than the sensor-space approach (in the distance and speed). We hypothesized that the disparity in terms of the decoding improvement might be due to the nature of the task as our decoder gives continuous output as opposed to the discrete output in Li et al. (2021).

We saw that *PCA* + *MRI* exhibited the highest median metrics more often than other approaches (correlation: horizontal position, distance, and speed; SNR: horizontal velocity, distance, and speed). Still, we cannot be decisive about which approach is the best among the source-space approaches because Mean + MRI, Mean, and PCA showed only slightly lower metrics than *PCA* + *MRI* in those cases (see Supplementary Table 1). The improvement of the *Mean* and *PCA* variations over *Sensor* was also not statistically significant, only in the case of SNR of horizontal velocity and vertical position that *LPP* showed significantly lower SNRs than *Sensor*. We observed slightly better performance in *PCA* and *Mean* variations by including the subject-specific head model over the template head model, but we found no significant difference between them. This means that the template head model might be sufficient for the decoding, which was reported similarly in Edelman et al. (2019).

The performance of the source-space approaches was also reflected in the decoding patterns (Figure 9). As the task in this study involved the visuomotor task, we would expect the activity in the regions to correspond to the parietofrontal network (Culham and Valyear, 2006; Marek and Dosenbach, 2018). In this case, the activity should be around SPL, similar to the source-space analysis in Kobler et al. (2018, 2020c), Martínez-Cagigal et al. (2020) and Mondini et al. (2020). The decoding patterns of *Mean*, *Mean* + *MRI*, *PCA*, *PCA* + *MRI* were similar such that the decoder utilized the information mainly in PCU, CU, and SPL, which is in line with the frontoparietal network (Culham and Valyear, 2006). The decoder trained with *LPP* utilized the information from the anterior part of superior frontal gyri (aSFG), while in *LPP* + *MRI*, the decoder relied on the information from SOG. The dominating activity in *LPP* variations could not be from artifacts because the same processed data was used for every approach, but this activity was only presented in *LPP* variations. As we know from the literature that aSFG and SOG would contribute less information than the areas around SPL, the lower decoding performance than the other approaches was expected. The decoding patterns in *LPP* that differ from *PCA* and *Mean* could be explained by the different optimization criteria between DR techniques because the optimizing criteria of *LPP* were to reduce the dimension while retaining the locality information across observations, while *PCA* tries to preserve the component that explained the variance of the data, and in the case of Mean, it simply calculates the averaged signals across voxels. Another interesting observation in the decoding patterns was that the decoding patterns in PCU and CU of the velocity kinematics at 0th lag were similar to the decoding patterns of the position kinematics at −6th lag (or at −300 ms) for *PCA* and *Mean* variations (see Supplementary Figures 1–4). The lagging behavior of the velocity was expected because of the temporal dependencies between the position and velocity, as discussed in Kobler et al. (2018) and Mondini et al. (2020). One limitation with the visualization of the decoding patterns with our approaches was that the patterns seem to be coarser than typical source localization results (i.e., every source in the same ROI has the same weight/color). The decoding was done directly in the source space so the decoding patterns were already in the source space, in contrast to sensor-space decoding that the decoding patterns had to be projected onto the source space. In the case of MRI variations, the decoding patterns seemed finer than the ICBM152 variations because of the smoothing effect from the projection of the subject-specific to the template head model before averaging. This effect could be seen in Figure 9 where the dark blue area in the MRI variations spreads out more than the ICBM152 variations. Another limitation was that we tried to define the ROIs similarly in both the MRI and ICBM152 variations such that the ROIs covered the same area, but it was not exactly the same, as seen in Figures 2C,D so that the differences in terms of decoding performance or the decoding patterns could also be from the slightly different ROIs.

Several studies utilized the signals in the source space rather than the sensor space, for example, in the MRCP detection (Li et al., 2021), motor imagery classification (Qin et al., 2004; Xygonakis et al., 2018; Edelman et al., 2019, 2016), and arm direction classification (Shenoy Handiru et al., 2017) where they reported higher decoding performance in comparison to the sensor space approach. However, these studies involved the discrete problem of classification rather than the continuous problem as a regression. A recent study (Sosnik and Zheng, 2021) investigated the source-space-based decoding in a similar scenario, where they tried to regress the velocity of arm joints in three dimensions. They reported a significantly lower overall correlation in the source-space decoding than in the sensor-space (0.28 and 0.25 in sensor and source space, respectively). Our results showed that the hand trajectory could be decoded in the source-space with slightly (but not statistically significant) higher correlations compared to the sensor-space signals (position: 0.310 and 0.313, velocity: 0.349 and 0.353, magnitude: 0.132 and 0.136 in sensor and source space, respectively). However, a direct comparison was not possible due to several differences in the experimental setup (e.g., 2D vs. 3D decoding), processing steps (e.g., decoding models, differences in how the source-space signals were extracted).

There are also some points that we would like to discuss beyond the results represented. First, the benefit of the source-space decoding over the sensor-space decoding, and second, the merit of continuous decoding in contrast to other control schemes in BCI. For the first point, we saw that the source-space decoding is on-par with the sensor-space decoding despite utilizing more computational resources. One may ask whether it is beneficial to continue with the source-space decoding. However, there might still be some room for improvement. We believed that the source-space decoding might be a promising direction as we could try to improve the decoding performance by using a more sophisticated way to solve the forward or inverse problems during the source localization. Also, we believed that the source-space might represent the common space among measurement units (e.g., across sessions for the same participant, or across different participants) and might play an important role in reducing the calibration which is a considerable concern in the BCI applications. Though, to confirm this point, we need to conduct further experiments. For the second point, even though the continuous decoding of hand kinematics showed moderate decoding accuracy, we believed that this control scheme feels more natural than classical BCI control schemes such as motor imagery or P300 BCIs (Müller-Putz et al., 2016). We also would like to move towards the usage in the end-users group such as people with limited hand functionality. To achieve this goal, we would like to further investigate the decoding not just in an executed hand movement condition as previously done but also in an attempted hand movement (Muller-Putz et al., 2021; Pulferer et al., 2021).

## Conclusion

In this study, we showed that the source-space decoding of the hand trajectory could be done with similar correlations as in the sensor space by performing the DR using PCA with eight components. We also observed no significant difference between using a template head model or a subject-specific head model. The group level decoding patterns revealed similar contributions from the brain regions in both the template and subject-specific head models. The proposed way of reducing the source-space signal in this study was simple because it involves applying the DR technique after the source-space transformation. However, more elaborate approaches that utilize the information from the training data during the source localization would be one of the promising directions of development in source-space decoding.

## Data Availability Statement

The raw data supporting the conclusions of this article will be made available by the authors, without undue reservation.

## Ethics Statement

The studies involving human participants were reviewed and approved by the Medical University of Graz. The patients/participants provided their written informed consent to participate in this study.

## Author Contributions

NS and GM-P conceptualized the idea, performed the proofreading, and finalized the manuscript. NS performed the analysis and wrote the initial draft. Both authors contributed to the article and approved the submitted version.

## Conflict of Interest

The authors declare that the research was conducted in the absence of any commercial or financial relationships that could be construed as a potential conflict of interest.

## Publisher’s Note

All claims expressed in this article are solely those of the authors and do not necessarily represent those of their affiliated organizations, or those of the publisher, the editors and the reviewers. Any product that may be evaluated in this article, or claim that may be made by its manufacturer, is not guaranteed or endorsed by the publisher.
