# Supplementary material for: Applying Dimensionality Reduction Techniques in Source-Space Electroencephalography via Template and Magnetic Resonance Imaging-Derived Head Models to Continuously Decode Hand Trajectories
Source: Front Hum Neurosci. 2022 Mar 24;16:830221. doi: 10.3389/fnhum.2022.830221 (PMC8988304; doi:10.3389/fnhum.2022.830221)
Supplement: Supplementary file 1 [file Data_Sheet_1.PDF]

Supplementary Material

1 Supplementary Tables

Supplementary Table 1. Group level median values of the correlations and SNRs, averaged over measurement runs. The table summarizes the boxplots in figure 8. The difference columns indicate the improvement of each approach to *Sensor*. The numbers marked in bold indicate the highest metrics among approaches.

Kinematic	Approach	Correlation	Diff. Corr.	SNR	Diff. SNR
Horizontal position	Sensor	0.3627		-1.3980	
	Mean	0.3839	0.0212	-1.5148	-0.1168
	PCA	0.3903	0.0276	-1.3064	0.0916
	LPP	0.3636	0.0008	-1.7070	-0.3090
	Mean+MRI	0.3877	0.0250	**-1.2552**	**0.1428**
	PCA+MRI	**0.3908**	**0.0281**	-1.2682	0.1298
	LPP+MRI	0.3643	0.0016	-1.7072	-0.3092
Horizontal velocity	Sensor	0.4073		-0.9902	
	Mean	0.4312	0.0239	-1.2296	-0.2394
	PCA	0.4289	0.0216	-1.0520	-0.0618
	LPP	0.3750	-0.0323	-1.3810	-0.3908
	Mean+MRI	**0.4318**	**0.0245**	-1.0117	-0.0215
	PCA+MRI	0.4299	0.0226	**-0.9863**	**0.0039**
	LPP+MRI	0.4079	0.0006	-1.4987	-0.5085
Vertical position	Sensor	**0.2875**		-2.4084	
	Mean	0.2674	-0.0201	-2.3694	0.0390
	PCA	0.2854	-0.0021	**-2.2154**	**0.1930**
	LPP	0.2624	-0.0251	-2.6015	-0.1931
	Mean+MRI	0.2837	-0.0038	-2.2452	0.1632
	PCA+MRI	0.2589	-0.0286	-2.3145	0.0939
	LPP+MRI	0.2814	-0.0061	-2.5688	-0.1604
Vertical velocity	Sensor	0.3248		**-1.4513**	
	Mean	0.3004	-0.0244	-1.5786	-0.1273
	PCA	**0.3295**	**0.0047**	-1.5360	-0.0847
	LPP	0.3090	-0.0159	-1.7739	-0.3226
	Mean+MRI	0.3059	-0.0189	-1.5713	-0.1200
	PCA+MRI	0.2995	-0.0253	-1.5266	-0.0753
	LPP+MRI	0.3215	-0.0033	-1.9875	-0.5362
Distance	Sensor	0.0873		-3.7628	
	Mean	0.0950	0.0077	-3.6334	0.1294
	PCA	0.1067	0.0194	-3.5644	0.1984
	LPP	0.0839	-0.0034	-3.8965	-0.1337
	Mean+MRI	0.1038	0.0165	-3.6062	0.1566
	PCA+MRI	**0.1091**	**0.0218**	**-3.5002**	**0.2626**
	LPP+MRI	0.0734	-0.0139	-4.1642	-0.4014
Speed	Sensor	0.1896		-3.2620	
	Mean	0.1952	0.0056	-3.2424	0.0196
	PCA	0.1930	0.0034	-3.1768	0.0852
	LPP	0.1388	-0.0508	-3.6529	-0.3909
	Mean+MRI	0.1805	-0.0091	-3.3081	-0.0461
	PCA+MRI	**0.1976**	**0.0080**	**-3.1370**	**0.1250**
	LPP+MRI	0.1359	-0.0537	-4.0134	-0.7514

2 Supplementary Figures

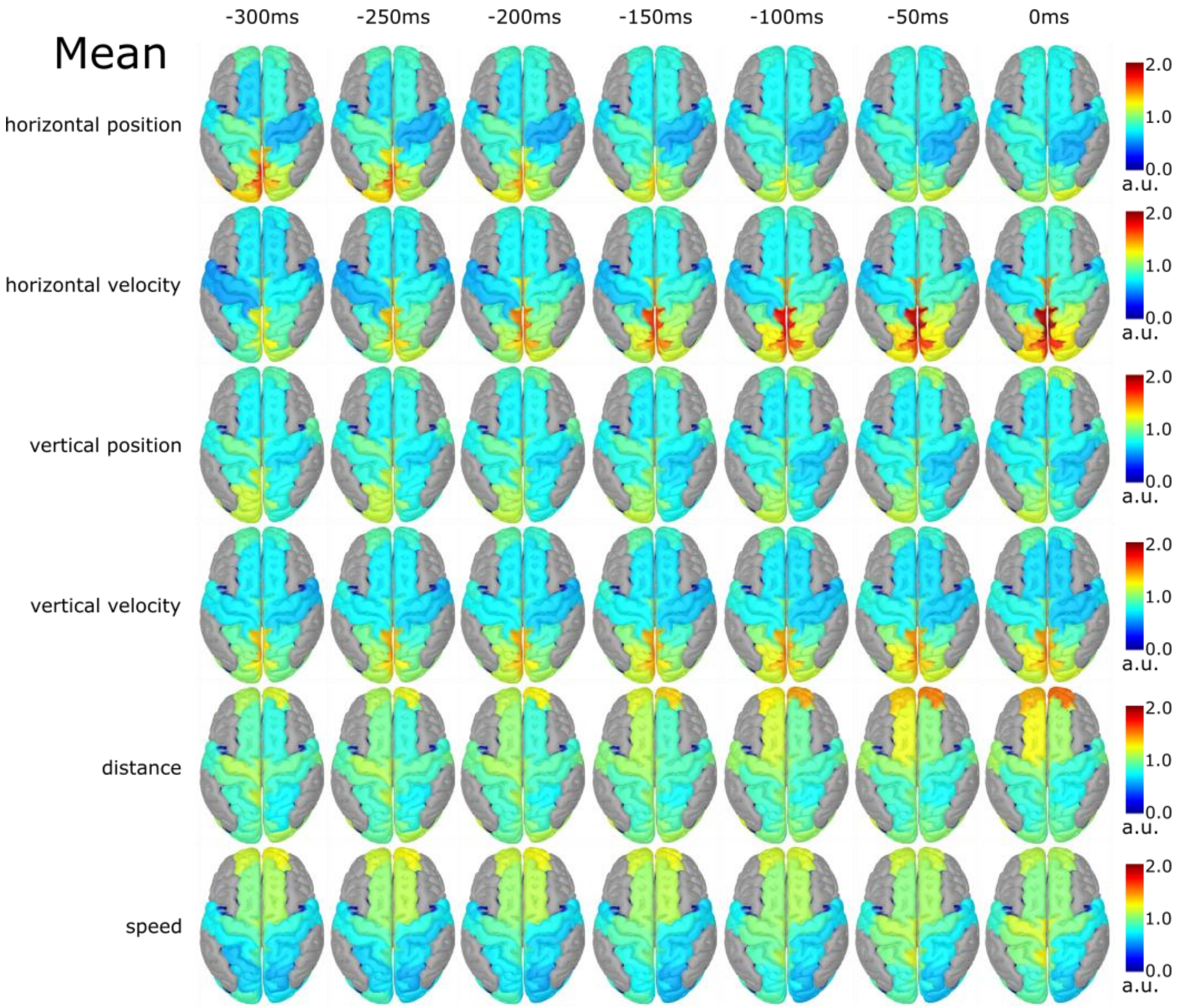

Supplementary Figure 1. The decoding pattern of *Mean* of the multilags source-space signals between -300ms to 0ms

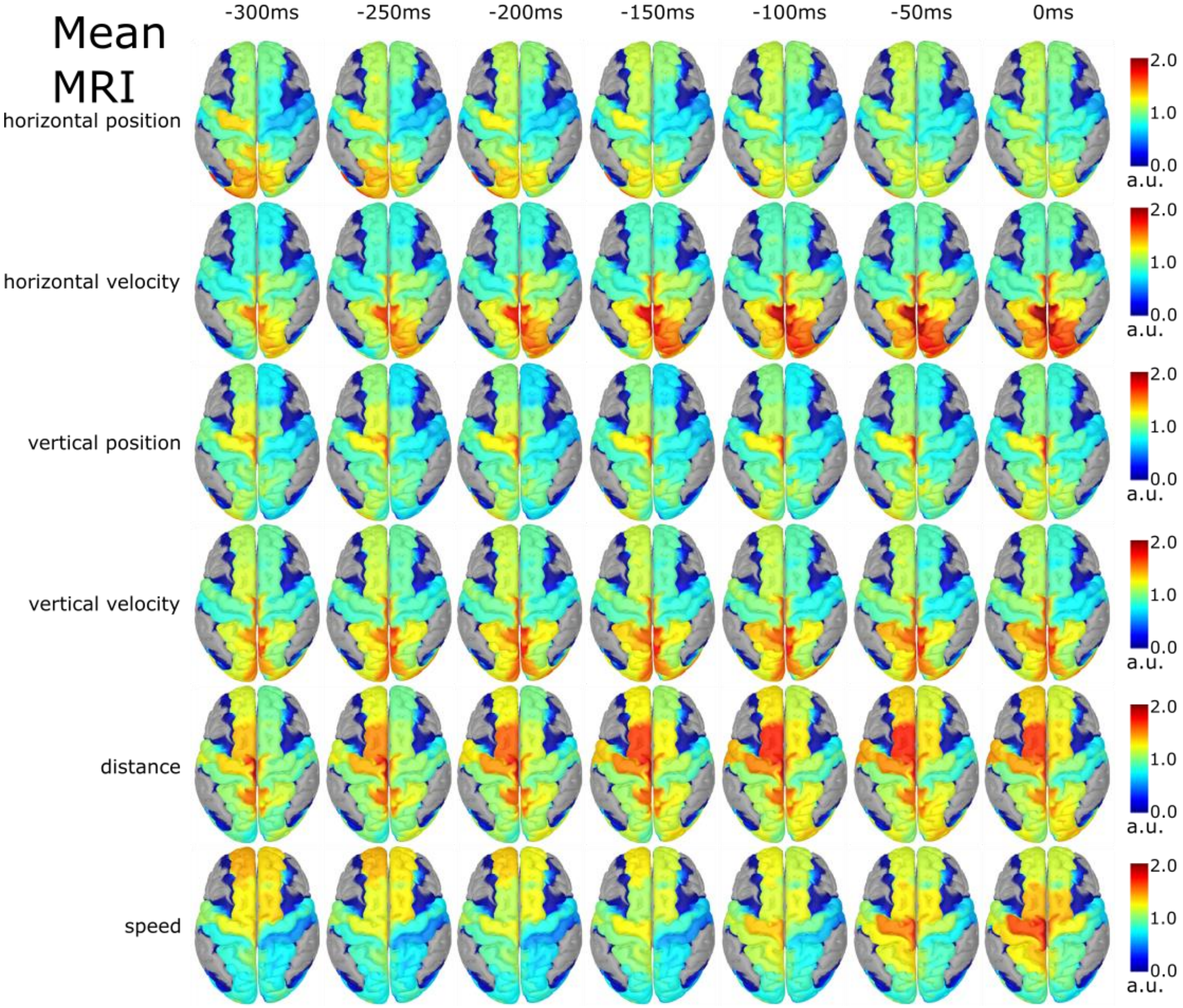

Supplementary Figure 2. The decoding pattern of *Mean+MRI* of the multilags source-space signals between -300ms to 0ms

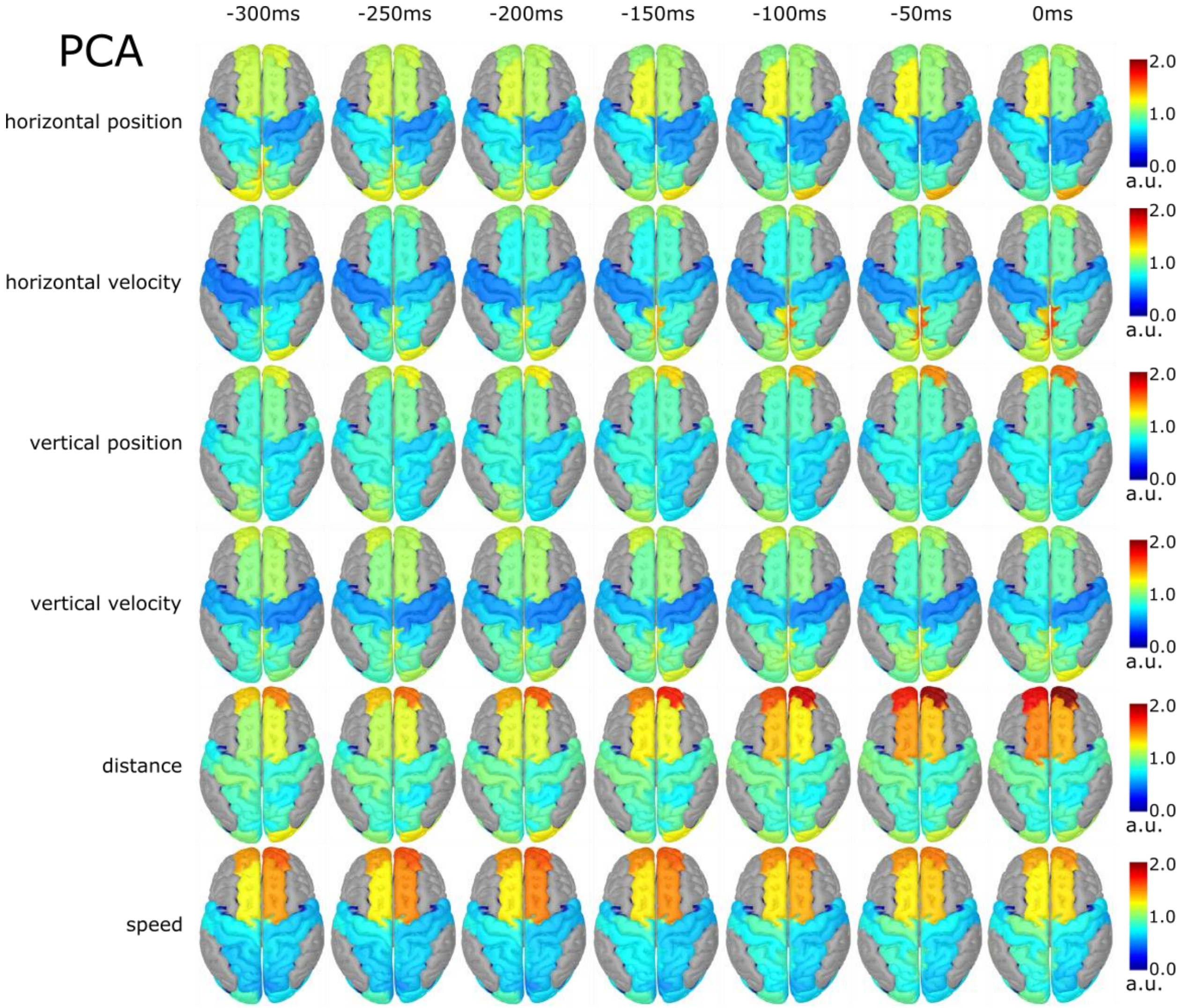

Supplementary Figure 3. The decoding pattern of *PCA* of the multilags source-space signals between -300ms to 0ms

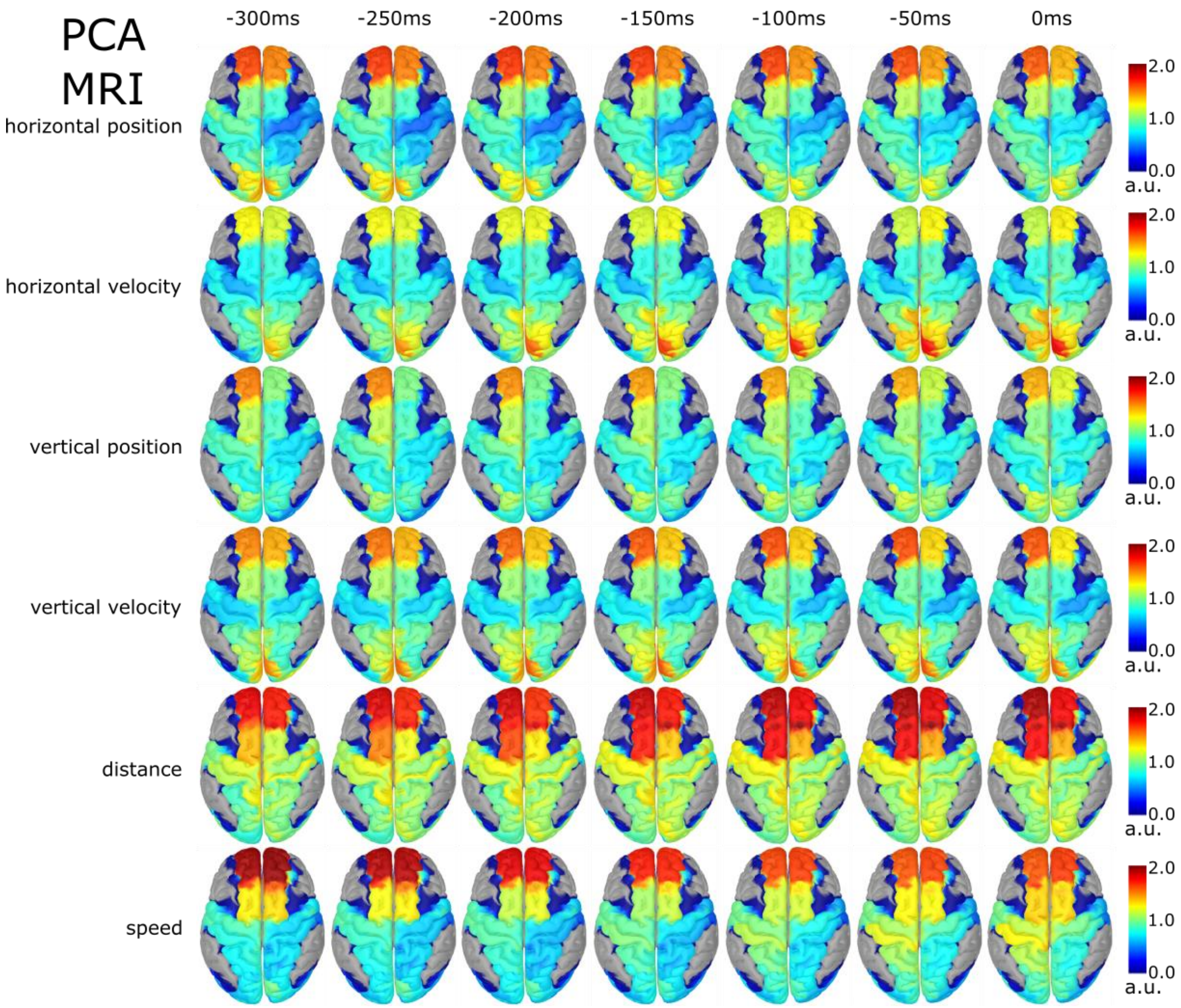

Supplementary Figure 4. The decoding pattern of *PCA+MRI* of the multilags source-space signals between -300ms to 0ms

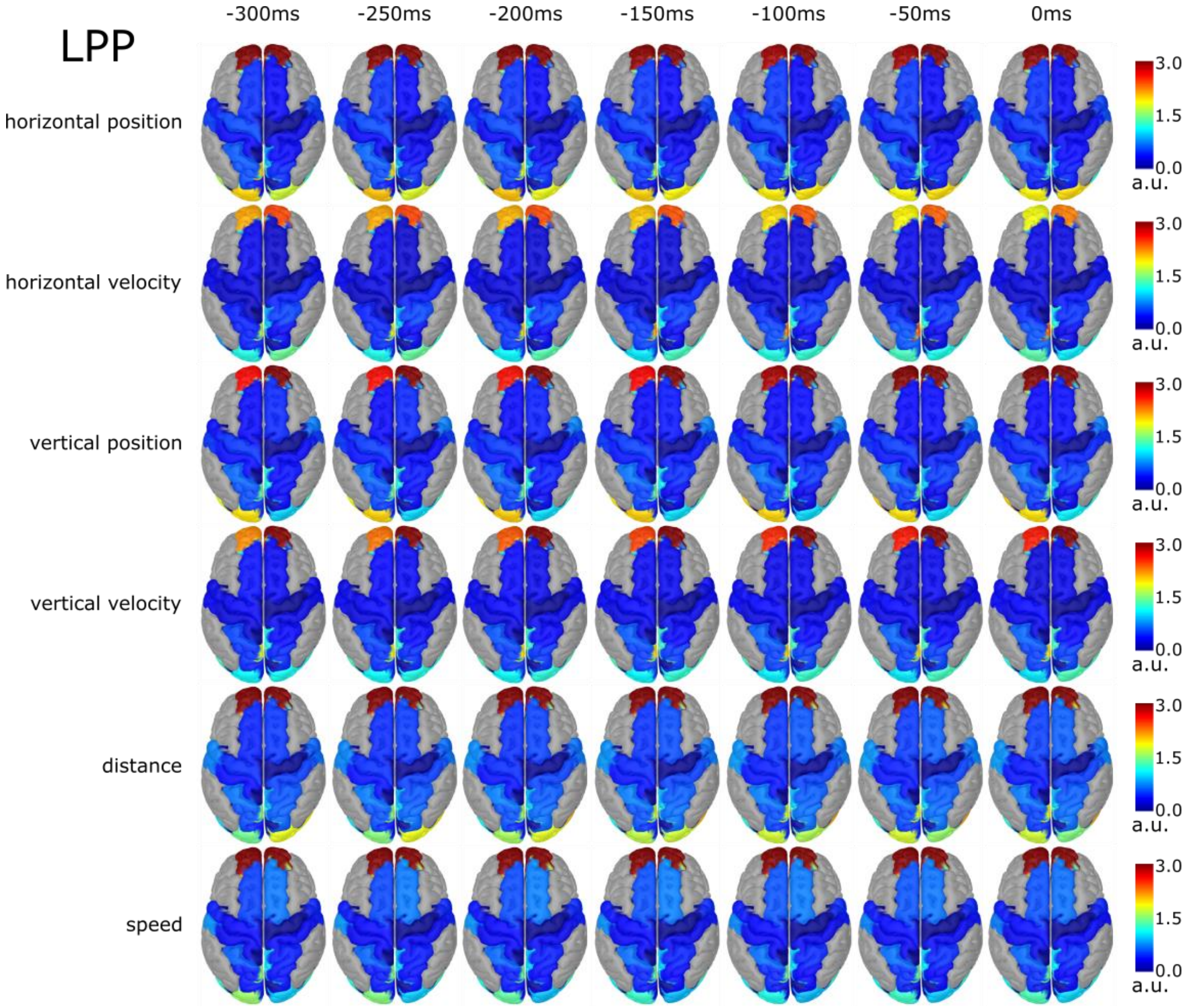

Supplementary Figure 5. The decoding pattern of *LPP* of the multilags source-space signals between -300ms to 0ms

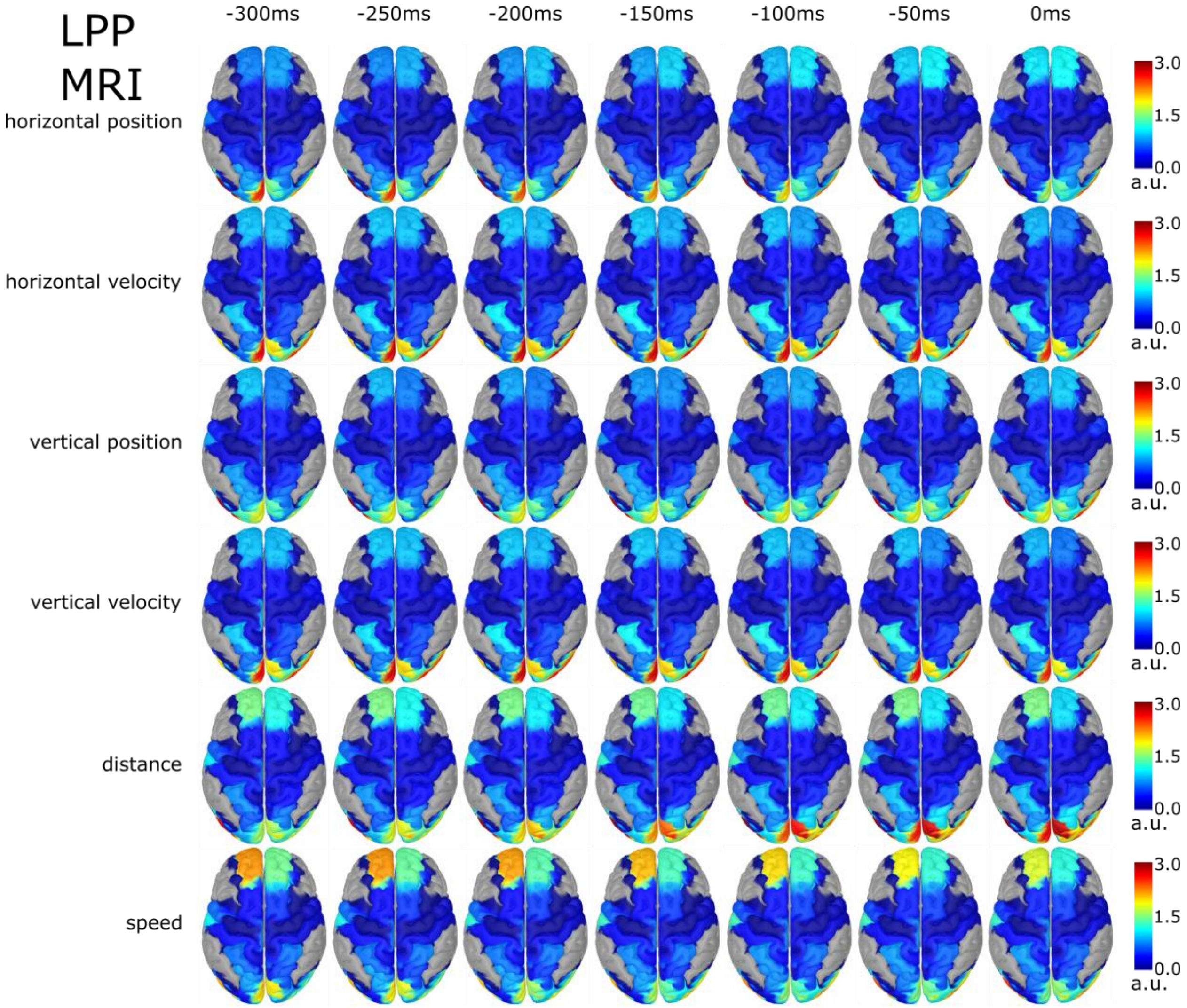

Supplementary Figure 6. The decoding pattern of *LPP+MRI* of the multilags source-space signals between -300ms to 0ms
